# Deep Learning in LncRNAome: Contribution, Challenges, and Perspectives

**DOI:** 10.3390/ncrna6040047

**Published:** 2020-11-30

**Authors:** Tanvir Alam, Hamada R. H. Al-Absi, Sebastian Schmeier

**Affiliations:** 1 College of Science and Engineering, Hamad Bin Khalifa University, Doha 34110, Qatar; halabsi@hbku.edu.qa; 2 School of Natural and Computational Sciences, Massey University, Auckland 0632, New Zealand; S.Schmeier@massey.ac.nz

**Keywords:** long non-coding RNA, lncRNA, lncRNAome, deep learning, machine learning, convolutional neural network, CNN, LSTM, Attention mechanism

## Abstract

Long non-coding RNAs (lncRNA), the pervasively transcribed part of the mammalian genome, have played a significant role in changing our protein-centric view of genomes. The abundance of lncRNAs and their diverse roles across cell types have opened numerous avenues for the research community regarding lncRNAome. To discover and understand lncRNAome, many sophisticated computational techniques have been leveraged. Recently, deep learning (DL)-based modeling techniques have been successfully used in genomics due to their capacity to handle large amounts of data and produce relatively better results than traditional machine learning (ML) models. DL-based modeling techniques have now become a choice for many modeling tasks in the field of lncRNAome as well. In this review article, we summarized the contribution of DL-based methods in nine different lncRNAome research areas. We also outlined DL-based techniques leveraged in lncRNAome, highlighting the challenges computational scientists face while developing DL-based models for lncRNAome. To the best of our knowledge, this is the first review article that summarizes the role of DL-based techniques in multiple areas of lncRNAome.

## 1. Introduction

The transcriptional landscape in eukaryotic organisms (e.g., humans) is now perceived as far more intricate than was originally thought [1] after the discovery that only about 2% of the genomic regions in humans encode for proteins, and the remaining sequences are non-coding regions that do not encode for proteins [2]. Since most of the human genome is transcribed, whether it encodes a protein or not, a major part of the human genome is pervasively transcribed into non-coding RNAs (ncRNAs). From this expanded view of ncRNAs, long non-coding RNAs (lncRNAs), which are more than 200 nucleotides in length, have recently been in the limelight due to evidence of linking mutations in their sequence to the dysregulation in many human diseases [3]. For example, genome-wide association studies (GWAS) have discovered that the long non-coding RNA (lncRNA) ANRIL is significantly associated with susceptibility to type 2 diabetes, intracranial aneurysm, coronary disease, and several types of cancers [3]. There are several mutations within the ANRIL gene body, as well as in its surroundings, that are correlated with a propensity for developing the above-mentioned diseases [3]. Another example of an lncRNA is Gas5, which is involved in susceptibility to auto-immune disorders [4] and could also act as a tumor suppressor in breast cancer [5]. Besides these examples, numerous other lncRNAs are involved in a multitude of human diseases. Interested readers may refer to the following articles to get a more detailed picture of the role of lncRNAs in different diseases [3,6,7,8].

In the early 1980s, scientists used to consider the hybridization of complementary DNA (cDNA) for cloning the genes and measuring their expression and tissue-specificity [9]. Initially, the efforts were focused on genes that were known to produce proteins. Then, the scientific community adopted the same approach for RNAs without considering their coding potential. Based on this approach, the first discovered lncRNA in a eukaryotic organism was H19. The intriguing factor about the discovery of H19 was the absence of being translated even though it had small open reading frame sequences in the gene body. Surprisingly, the transcripts of H19 showed similar characteristics to those of messenger RNAs (mRNA) in terms of splicing, polyadenylation, localization in the cytoplasm, and its transcription by RNA polymerase II [10]. From the roster of the earliest discovered lncRNAs, X-inactive-specific transcript (XIST) is among the most well-studied lncRNAs due to its role in the X-chromosome inactivation (XCI) phenomena [11]. The loci of XIST was discovered in the early 1990s, and it showed very low expression levels in mouse undifferentiated embryonic stem (ES) cells for both males and females [12,13]. Since the pioneering discoveries of H19 and XIST, the view on non-coding genes in the scientific community has changed completely and has rejuvenated the efforts to discover and characterize novel non-coding RNAs. Specifically, studying lncRNAs has increased dramatically. Additionally, advancement in next-generation-sequencing technology enabled the discovery of many functional lncRNAs in the non-coding regions of the human genome. LncRNAs, despite being considered to be junk DNA regions for approximately the last twenty years [14], are now recognized as being pervasively transcribed, and non-coding RNA transcriptomes (specifically lncRNAs) have become a major field in biomedical research.

The pervasive nature of the transcriptomes in humans [15] and mice [16] has also been highlighted by the Functional Annotation of the Mammalian Genome (FANTOM) consortium in the largest collection of functional lncRNAs, with over 23,000 lncRNA genes [17]. GENCODE [18] v25 provides a list of ~18,000 human lncRNA genes. MiTranscriptome has collected 58,548 lncRNA genes [19], however, it is unclear if all of them are functional. From this, we can observe that the discovery of novel lncRNAs is becoming a regular occurrence, and the catalogue of lncRNAs is constantly growing. Therefore, it is of interest to analyze this large, versatile, and dynamic collection of lncRNAs in a systematic fashion using state-of-the-art computational techniques to derive novel hypotheses, discover unanticipated links, and make proper functional inferences [20]. Machine learning (ML)-based methods are well suited for lncRNA research, since ML-based techniques can generate insights and discover new patterns from the growing number of lncRNA repositories.

Though ML-based methods are applicable to different types of data, the performance of ML-based models depends on the representation of the data. The quality of data representation and the relevance of the data to a particular problem affect the performance of ML-based models. Deep learning (DL), a sub-field of ML, can address this issue by embedding the data for the model to yield end-to-end models [21]. DL, a biology-inspired neural network [22], uses multiple hidden layers and is considered to be among the best paradigms for classification and prediction in the ML field [23]. In the past ten years, DL-based models have achieved tremendous success in computer vision [24], machine translation [25], and speech recognition [26]. The main reason for their success is the unprecedented availability of massive volumes of data, improvement of computational capacity, and the advancement of sophisticated algorithms [27,28]. The enormous amount of biological data, which was once considered to be a big analysis challenge, transformed into an opportunity for biomedical researchers [29]. DL-based methods have now been successfully applied in the genomics research domain [21].

Considering the functionally diverse role of lncRNA in different human biological processes and diseases and the extreme capacity of DL to identify informative patterns from big data, we reviewed how DL has facilitated the discovery of the role of lncRNAs in different human diseases and the underlying mechanism in a data-driven fashion. To the best of our knowledge, this article is the first to summarize the contribution of DL in multiple research domains of lncRNAome.

We organized this article in the following way. We first introduce a primer on DL techniques that were successfully applied in different lncRNAome-related problems. Then, we highlight the DL-based methods that have been successfully applied in several lncRNA-related research problems. We continue by discussing potential issues that might be encountered by researchers while implementing DL-based solutions for lncRNAome and possible resolutions. Finally, we conclude by discussing the perspectives of DL methods in lncRNAome research areas.

## 2. Summary of Deep Learning Techniques that Are Applied in lncRNAome-Related Research Problems

In this section, we provide a brief description of the deep learning (DL) models that have successfully been used in the modeling of lncRNAome-related research problems.

### 2.1. Neural Network

**Figure 1:**
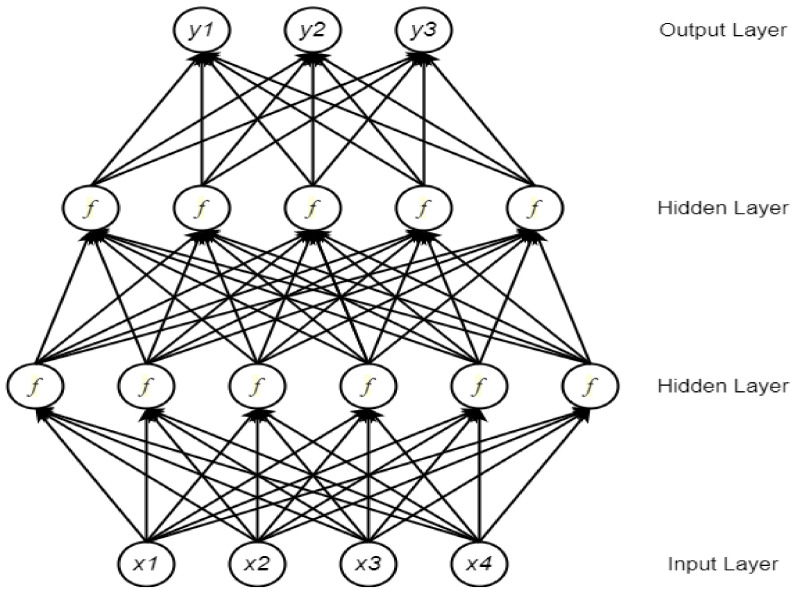
A neural network (NN) with four inputs and two hidden layers (adopted from [32]). x_i_ represents an input feature for the network, and y_i_ represents an output class label.

A neural network (NN) comprises multiple processing components, or parts, that are joined to form a network with adjustable weighting functions for each input. The NN components are organized in several connected layers. Typically, there are three types of layers in a NN: input layer, hidden layer(s), and output layer [30]. The input layer considers data to be fixed-size input values and presents them through the hidden layers inside the network. To propagate from one layer to the next, a weighted sum of the inputs from the previous layer is passed through a non-linear function. Finally, a fixed-sized output is generated through the output layer. Currently, the most popular function for the hidden layers is the rectified linear unit (ReLU) [31]. Depending on whether a task is a binary or a multi-class classification problem, a Sigmoid or a Softmax function is used at the output layer. Figure 1 shows a typical NN architecture for vector inputs.

### 2.2. Deep Neural Network

A deep neural network (DNN) is a neural network that has multiple hidden layers. These multiple learning layers allow for learning representations of data that have many levels of abstraction, which leads to improvements in model performance in many applications such as object detection, speech recognition, and many more [31].

### 2.3. Deep Belief Network

**Figure 2:**
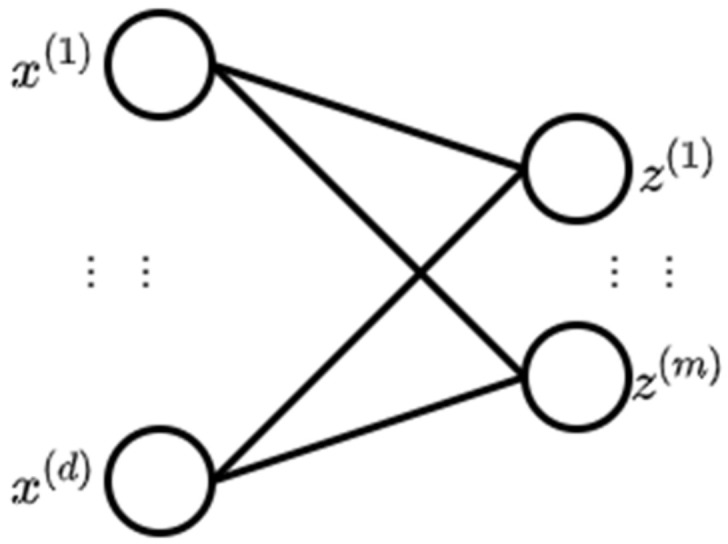
Restricted Boltzmann machine (RBM) (adopted from [34]).

A deep belief network (DBN) is a network of multiple layers where each layer consists of a restricted Boltzmann machine (RBM) with a classifier in the last layer [33]. An RBM is a neural network with two layers where the left layer is the visible layer and the right layer is the hidden layer (Figure 2) [34]. The visible layer represents a less abstract form of the raw data where the hidden layer is trained to represent more abstract features [35].

**Figure 3:**
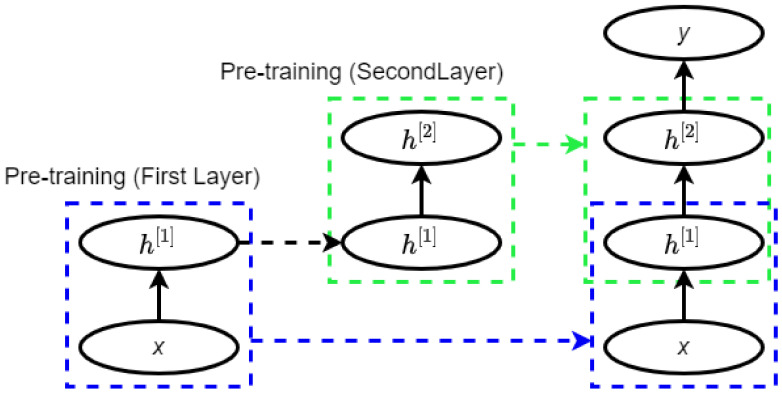
Pretraining of a deep belief network (DBN) (adopted from [36]).

In DBN, learning happens in one layer at a time. When an RBM layer has learned its feature activation, it is issued as input to the following RBM layer and so on. Repeating the trainin, layer-by-layer oftentimes leads to a DL model [36]. Figure 3 shows the pretraining of a DBN.

### 2.4. Convolutional Neural Network

**Figure 4:**
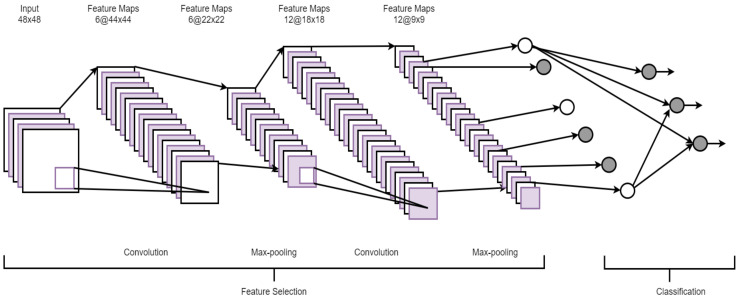
An architecture of a convolutional neural network (CNN) (adopted from [39]).

A convolutional neural network (CNN) is a hierarchical model that learns patterns at multiple layers using a series of 1D, 2D, or 3D convolutional operations [31]. A CNN usually consists of multiple layers, namely, a convolutional layer, a non-linearity layer, a pooling layer, and a fully-connected (FC) layer(s) [37]. However, it is important to stress that all of these layers are not mandatory to build a CNN. Multiple stages of these layers are followed by conventional fully connected layers. A set of filters is used in the convolutional layer to extract spatial features from the input data and the pooling layer reduces the dimension of the data after convolution steps. Since FC layers have a large number of parameters, making it harder to train the network, a new type of layer, global average pooling [38], can be applied directly to the output of the final convolution layer, eliminating the need for the FC. Since pooling operations might discard useful information from the input, strided convolution has recently been researchers’ preference. Figure 4 shows the architecture of a typical CNN.

### 2.5. Graph Convolutional Network

**Figure 5:**
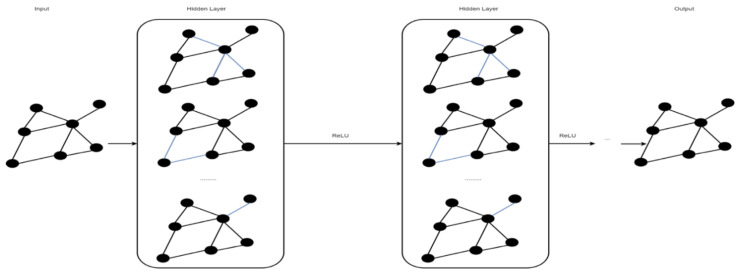
A graph convolutional network (GCN) (adopted from [41]).

A graph convolutional network (GCN) is a type of convolutional neural network that works on graphs [40]. A GCN’s input is a graph with labeled nodes, and the output is all the input graph’s nodes labeled as predictions. Similar to CNNs or multi-layer perceptrons (MLP), for any input, a GCN learns new features that later become inputs to the classifier over multiple layers. Unlike an MLP, at the beginning of each layer, a GCN averages the features of each node with feature vectors in the neighborhood [40]. Figure 5 shows an example of a GCN.

### 2.6. Generative Adversarial Network

**Figure 6:**
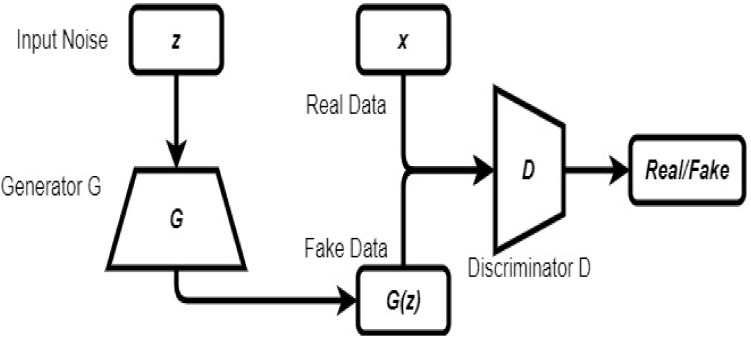
Architecture of a generative adversarial network (GAN) (adopted from [42]).

A generative adversarial network (GAN) is a model that comprises generative and discriminative models. Both models are trained in an adversarial manner where the generator generates fake inputs that seem real, and the discriminative model tries to classify inputs as either real or fake [42]. In this model, the training process for the generator is to maximize the probability of the discriminator making a mistake [43]. This model can be used in applications related to data synthesis, classification, and image super-resolution [42]. Figure 6 shows an architecture of a GAN.

### 2.7. Autoencoder

**Figure 7:**
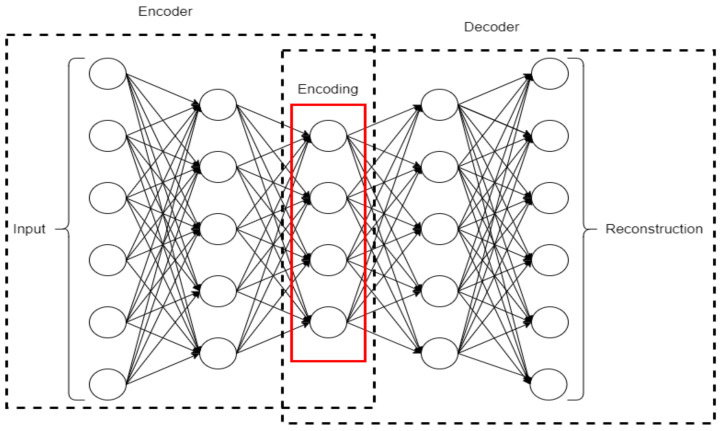
Architecture of an autoencoder (AE) (adopted from [45]).

An autoencoder (AE) is a type of neural network that learns the latent, lower-dimensional representation of input variables by passing the input variables through a bottleneck layer in the middle of the network and reconstructing the input variable at the output layer [44]. The loss function used in training this network penalizes the input reconstruction error. After convergence, the trained network can be used for input reconstruction with minimal noise [45]. One of the advantages of an AE is that it can be used in learning a lower-dimensional representation of input data with low reconstruction error even when it spans a non-linear manifold in a feature space. Figure 7 shows an architecture of an AE.

### 2.8. Recurrent Neural Network

**Figure 8:**
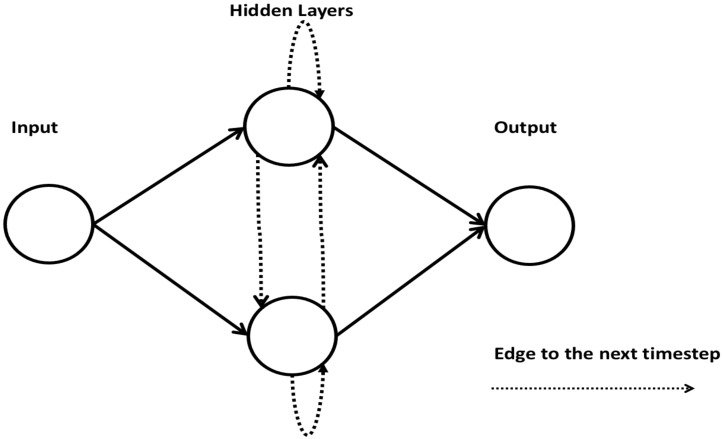
A simple architecture of an RNN.

A recurrent neural network (RNN) is made of artificial neurons with one or more feedback loops. A simple RNN architecture consists of an input layer, multiple recurrent hidden layer(s), and an output layer [46]. An RNN constructs recurrent connections over a period of time, and activation from time steps is stored in the internal memory of the network. This makes an RNN suitable for applications related to time series and sequential data [47]. Figure 8 shows an architecture of an RNN.

**Figure 9:**
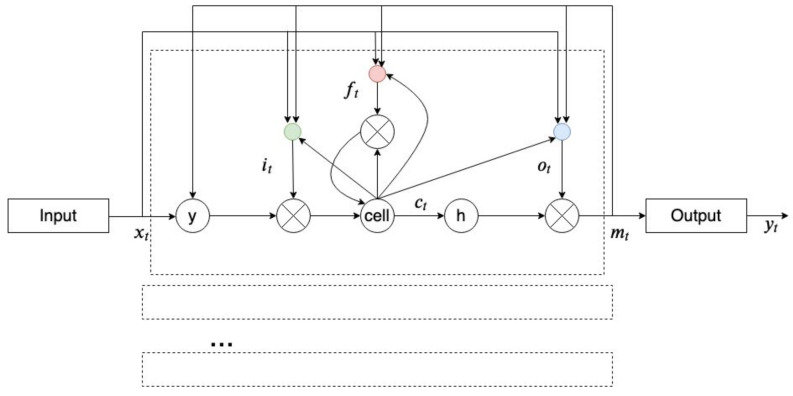
A long short-term memory (LTSM) architecture (adopted from [49]).

A long short-term memory (LSTM) is a type of RNN that reduces the effects of vanishing and exploding gradients (which is a drawback of an RNN that happens during the training of an RNN) in an RNN. LSTM changes the structure of hidden units from “sigmoid” or “tanh” to memory cells where gates control inputs and outputs and maintain extracted features from preceding timesteps [48]. Figure 9 shows an LTSM memory block.

**Figure 10:**
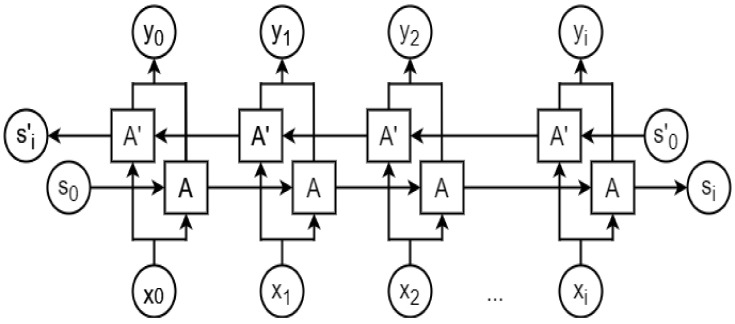
A bidirectional LSTM (BLTSM) architecture. A and A’ represent an LSTM cell propagating data dependency in forward and reverse directions, respectively. x_t_ and y_t_ are input and output at timestep t from each LSTM cell, respectively. S_0_ and S’_0_ denote the initial states, whereas S_i_ and S’_i_ denote the final states.

A bidirectional LSTM (BLSTM) is a variation of an RNN [50] that runs in both forward and backward directions, where the output from a cell depends on all the previous (forward direction) and future (backward direction) timesteps. A BLSTM has been found to perform better than a unidirectional LSTM if the output at a timestep depends on both past and future inputs. Figure 10 shows a typical BLTSM network structure.

### 2.9. Attention Mechanism (AM)

**Figure 11:**
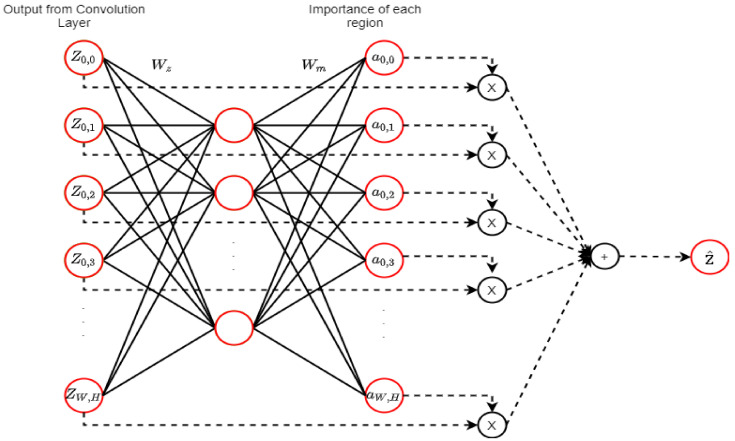
An attention mechanism (AM) (adopted from [51]). $Z_{i,j}$ denotes the output map from the middle of the convolution layer of a network. The map is propagated to the next layer of the network, and the AM calculates the weighted average of $Z_{i,j}$ as $\hat{Z}$. The fully connected layer calculation is represented by the straight lines, and the weighted average calculation is represented by dashed lines. The neural network is utilized by the AM to estimate $a_{i,j}$ and the importance of each $Z_{i,j}$.

An attention mechanism (AM) is a DL technique that was first introduced for language translation and performance enhancement that occurs by selecting significant features dynamically [51]. Figure 11 shows the attention mechanism in CNN that optimizes the weights and the biases to ensure the selection of important features in each region.

## 3. Summary of the lncRNAome Research Domains Where Deep Learning-Based Techniques Have Made Significant Contributions

Advances in next-generation sequencing techniques have afforded researchers the opportunity to study a plethora of novel lncRNA transcripts from multiple cells and tissues [17]. The state of lncRNA discovery and lncRNA annotation is still in its infancy. Several research groups are currently discovering new lncRNAs and applying different ML-based techniques to study different properties and functions of lncRNAs. In this section, we highlight different fields in the lncRNA research domain where DL-based techniques have been successfully used. An overview is given in Table 1.

### 3.1. LncRNA Identification

There are many existing methods for recognizing lncRNA transcripts which were developed based on shallow learning. For example, Lia et al. developed a tool called PLEK to recognize lncRNAs based on improved k-mer schemes [69]. Sun et al. developed the CNCI tool to distinguish lncRNA transcripts from protein-coding transcripts using the intrinsic composition of sequences [70]. An updated version of CNCI, called CNIT, which can provide the same solution with higher accuracy and faster speed has been produced [71].

Recently, due to the advancement of DL techniques, a lot of work has been published focusing on the identification of lncRNAs using DL-based techniques. For example, Tripath developed DeepLNC, a DNN-based network that uses k-mers (k = 1,2,3,4,5) from sequences as a feature set to distinguish lncRNA transcripts from mRNA transcripts [55]. Baek et al. developed lncRNAnet [52], which can be considered among the best of the performing models [72] for distinguishing full-length lncRNA transcripts from protein-coding transcripts. LncRNAnet used an RNN for sequence modeling and a CNN for the detection of stop codons to capture the open reading frame information. Yang et al. developed LncADeep, which can identify both partial and full-length lncRNA transcripts [53]. LncADeep incorporates different hand-curated features such as coding sequence (CDS) length, hexamer score, Fickett nucleotide features, etc. for developing a DBN-based model. In another recent publication, Liu et al. used k-mer embedding vectors for the sequences as input features and built the DL-based architecture using BLSTM and CNN [54]. Han et al. proposed an integrated platform for lncRNA recognition, which uses a sequence, structure, and physicochemical properties of sequences [73]. Interested readers may consult the review by Amin et al., which summarizes different DL-based methods that have been used to classify non-coding RNAs [72]. Table 2 provides a summary outcome from the articles that considered DL-based techniques to identify lncRNAs in multiple species.

As mentioned at the beginning of this section, many tools such as PLEK [69], CNCI [70], CNIT [71], etc. exist, and all of them were developed considering hand-curated features using traditional ML models for non-coding RNA identification. Interestingly, all the DL-based methods highlighted in Table 2 evaluated their proposed models against the traditional ML models and outperformed them for lncRNA identification, indicating the superiority of DL-based models over traditional ML models for this task.

### 3.2. Transcriptional Regulation of lncRNAs

To date, ML-based techniques have been used to detect underlying patterns in the promoter regions of lncRNAs and protein-coding genes [56,74,75]. Using an ML-based approach, Alam et al. showed that there are different sequence-specific patterns in the promoters of lncRNAs compared to the promoters of protein-coding genes. They also identified the list of transcription factors (TFs) that are involved in the transcriptional regulatory patterns specific to lncRNAs. Recently, Alam et al. developed a DL-based architecture, DeepCNPP, to distinguish the promoters of lncRNAs from the promoters of protein-coding genes ([56,74]. DeepCNPP was built using a CNN-based architecture and outperformed the existing models used for the same purpose. Alam et al. also developed a model, DeePEL, to distinguish between the transcription regulatory program of promoter-originated lncRNAs (p-lncRNA) and enhancer-originated lncRNAs (e-lncRNA) [57]. Table 3 provides a summary outcome from the articles that considered DL-based techniques to demystify the transcription regulation program for lncRNAs.

It is important to emphasize that the previous model [75] used for distinguishing the promoter of protein-coding genes and lncRNA genes incorporated hand-curated features based on the sequence of promoters, transcription factor binding sites at the promoter regions, CpG islands, repetitive elements, and epigenetic marks to achieve 81.69% accuracy on the classification task. On the other hand, the DL-based model, DeepCNPP [56], outperformed the previous model with 83.34% accuracy considering only the sequence-related information from the promoter of lncRNA genes.

### 3.3. Functional Annotation of lncRNAs

The functional annotation of lncRNA is a challenging task. There are many knowledge bases that collect the functionality of lncRNA based on the expression and/or the regulatory elements (transcription factors, transcription co-factors [76]) that are involved in their transcriptional regulation [20]. Some attempts to extract the known functionality of lncRNAs by literature mining have also been made [77].

Yang et al. developed LncADeep, a DNN-based architecture to infer the function of a lncRNA based on its interacting protein partners [53]. In lncADeep, Yang et al. used several sequence-and structure-related features from both lncRNA and proteins. These features were then fed into a DNN to predict lncRNA-protein interactions. To infer the function of lncRNAs, the authors used the Kyoto Encyclopedia of Genes and Genomes (KEGG) [78] and the Reactome [79] pathways enrichment of the predicted proteins. Since proteins usually work as functional modules [80], the authors also inferred the functional modules of lncRNAs based on interacting protein partners.

### 3.4. Predicting lncRNA Subcellular Localization

Cao et al. proposed an ensemble-based classifier to predict the location of lncRNAs in five subcellular locations: cytoplasm, cytosol, nucleus, ribosome, and exosome, yielding an overall performance accuracy of 59% [52,81]. Recently, Gudenas and Wang proposed the first DL-based localization predictor for lncRNAs. A DNN built only from sequence features is used to predict the subcellular localization of the lncRNAs, distinguishing between lncRNAs located in the nucleus and cytosol [58].

### 3.5. Predicting lncRNA–Protein Interactions

RNA binding proteins (RBP) play important roles in different biological processes [82] and are shown to be involved in different diseases, one of which is cancer [83]. With the advancement of sequencing technologies, RBP can be verified using cross-linking immunoprecipitation sequencing (CLIP-seq) [84]. However, these experiments are time-consuming and expensive. As an alternative, we can adopt a fast and affordable in silico approach using ML techniques for predicting RBP [85].

Many state-of-the-art tools for predicting lncRNA-protein interactions exist, such as lncPro [86], RPI-Pred [87], RPISeq-RF [88], etc., which were developed considering hand-curated features using traditional ML models. Among these tools, RPISeq-RF performed best for the task of lncRNA–protein prediction in many benchmark datasets [62]. Recently, DL-based architectures were used to predict lncRNA–protein interactions. For example, IPminer [59], RPI-SAN [60], and BGFE [61] are the tools where stacked auto-encoder networks were used to capture the important features of sequences, and then the learned features from the sequence were fed into random forest models to predict lncRNA-protein binding. Peng et al. developed a tool, RPITER [62], where they used stacked autoencoders and CNN to fit the k-mer sequence features and structure information from the RNA and protein.

Current methods have successfully predicted ncRNA and protein interactions with reasonably high accuracy, but most of the models were trained and tested on only small benchmark datasets mainly derived from ncRNA–protein complexes in a protein–RNA interaction database [89] or Protein Databank (PDB) [90]. Thus, there is a need for improving the generalization capability of these models. Interested readers may consult the review by Zhang et al. [91] for more details. Table 4 provides a summary outcome from the articles that considered DL-based techniques to predict lncRNA-protein interactions.

For lncRNA-protein interactions, multiple benchmark datasets exist (see Table 4) but there is no clear winner from the DL models (see Table 4) that performed the best in all benchmark datasets. For all benchmark datasets, there exists at least one DL-based model that outperformed the traditional ML-based models for the lncRNA–protein interaction prediction task. From the pool of conventional ML-based models, RPISeq-RF performed at a similar level of accuracy to the DL-based models in a few benchmark datasets [62]. Interested readers are encouraged to read the article by Yi et al. for more details [60].

### 3.6. Predicting lncRNA–miRNA Interactions

LncRNAs and microRNAs (miRNAs) interact with each other to form a complex regulatory network for controlling gene expression. Through this multi-level gene regulation (either transcriptional, post-transcriptional, or post-translational level), these two families of non-coding RNAs (miRNA and lncRNA) are involved in multiple aspects of cell cycles (e.g., cell division, cell differentiation, apoptosis). Recently, we witnessed an exponential growth of expression profiling of lncRNAs in different diseases and conditions, but information regarding lncRNA–miRNA interactions is still rare [92,93]. Huang et al. proposed the first large-scale lncRNA–miRNA predictive model using a network diffusion method on sequence information, expression profiles, and biological function ([93,94]). Similarly, Huang et al. proposed GCN-based model, graph convolution for novel lncRNA–miRNA interactions (GCLMI), to predict lncRNA–miRNA interactions [63]. Based on the proposed model, which combines graph convolution and an auto-encoder, Huang et al. found that the area under the curve (AUC) for the predictor was around 0.85, indicating that DL-based methods are important contributors in this research field.

### 3.7. Predicting lncRNA–DNA Binding

Prediction of lncRNA and DNA binding is a relatively new field of research. Until now, computational prediction of lncRNA–DNA interactions has received relatively little attention from the scientific community working in lncRNAome [95]. We did find several tools that assessed the triple helix formation of RNA–DNA interactions, namely Triplex [96], Triplex Domain Finder [97], Triplexator [98], Triplex-Inspector [99], and LongTarget [100].

Recently, Wang et al. proposed a DL-based model using different combinations of CNN and LSTM to predict the genome-wide DNA binding sites for twelve lncRNAs based on ChIRP-seq experimental data [64]. In that study, Wang et al. considered the best performing model to have two CNN layers and 32 kernels in each layer. The authors also concluded that LSTM-based models did not perform well, since long-range dependence along sequences is not necessary for lncRNA-DNA binding.

### 3.8. Predicting lncRNA-Disease Associations

There are many existing methods (e.g., Ping’s method [101], LDAP [102], SIMCLDA [103], MFLDA [104]) that have incorporated hand-curated features into traditional ML-based models to infer lncRNA–disease associations. Ping’s method and LDAP both consider similarity measures between lncRNAs and diseases to infer lncRNA-related diseases. Ping’s method also incorporates the topological information from the bipartite graph of the lncRNA–disease network to achieve better results than LDAP. On the other hand, SIMCLDA incorporates features from lncRNAs based on the Gaussian interaction profile kernels from lncRNA–disease interactions. SIMCLDA also incorporates features from diseases based on the Jaccard similarity of ontologies associated with diseases. Ping’s method and LDAP both performed better than SIMCLDA in benchmark datasets for multiple diseases [65]. MFLDA introduced a matrix factorization-based fusion model to predict lncRNA–disease associations. However, the performance of MFLDA was not as high compared to Ping’s method, LDAP, or SIMCLDA, as similarities between lncRNA and diseases were not incorporated into MFLDA [66].

Recently, Xuan et al. published a DL-based model called CNNLDA, a dual CNN with attention mechanisms for predicting lncRNA–disease associations [66]. CNNLDA integrates multiple sources of data considering similarities between diseases, similarities between lncRNAs, lncRNA–disease associations, disease–miRNA associations, and lncRNA–miRNA interactions under a single platform to outperform many of the state-of-the-art methods for predicting disease-related lncRNAs. Xual et al. also proposed another deep architecture, GCNLDA, which combines GCN and CNN to infer lncRNA–disease associations [65]. Hu et al. proposed NNLDA, a CNN-based DL architecture, that is used to predict the role of lncRNA in different diseases [67]. According to the authors, NNLDA was the first algorithm that considered deep neural networks for predicting lncRNA–disease associations. Table 5 provides a summary outcome from the articles that considered DL-based techniques to predict lncRNA–disease associations.

Compared to the traditional ML-based models (e.g., Ping’s method [101], LDAP [102], SIMCLDA [103], and MFLDA [104]), the DL-based models in Table 5 hugely improved the prediction of lncRNA–disease association. For example, CNNLDA outperformed Ping’s method, LDAP, SIMCLDA, and MFLDA by 8.05%, 8.85%, 20.6%, and 32.6%, respectively, in terms of AUC [66]. This clearly indicates the major contribution that DL-based models have made in the prediction of lncRNA–diseases associations.

### 3.9. Cancer Lassification

Mamun and Mondal proposed DL-based approaches to classify eight different cancer types using lncRNA expression profiles (RNA-seq) [68]. The authors discovered lncRNA expression to be a better signature compared to mRNA expression for classifying cancer types. Using four different types of deep neural networks (MLP, LSTM, CNN, and deep autoencoder (DAE)), the proposed models achieved an accuracy ranging from 94% to 98%.

## 4. Challenges for Deep Learning in lncRNA Research

In this section, we highlight some of the frequently encountered problems when building DL-based models for lncRNAome. We also briefly describe the problems and provide some recommendations to circumvent the issues.

### 4.1. Required Data Set Sizes

DL-based methods are most successful in supervised learning setups, where a sufficient number of samples are available for training the deep network. As a criterion, the number of training samples is expected to be as high as the number of total model parameters, although some regularization techniques can be used to avoid overfitting in cases of data scarcity [107]. LncRNAs are notoriously difficult to analyze, since their expression is low and cell-specific, making the number of lncRNAs from different cells and tissues available generally low. For image-based analysis, the training set can be augmented by different techniques such as rotation, scaling, or cropping [24]. However, for genomic sequences, the techniques are of a different type. For example, in the lncRNA–DNA binding prediction problem, Want et al. augmented the data by applying a random shift of genomic sequences either in the left or the right direction within a base pair range of 10 to 40 [64].

### 4.2. Imbalanced Datasets

Biological data are mostly imbalanced for training ML-based models [108]. There are many bioinformatics research problems where there is a need for handling such imbalanced data carefully, such as splice site predictions [109], poly (A) site predictions [110], protein–protein interaction motif findings [108], etc. Using imbalanced data for training DL-based models may result in undesirable or misleading results. To handle this issue, we need to follow specific criteria. First, we need to avoid using accuracy as an evaluation metric for models because accuracy is a misleading parameter for evaluating the performance of a model that uses imbalanced data. Instead of accuracy, we may use the area under the precision-recall curve (AUPRC), Matthews correlation coefficient (MCC), or F1-measure as a criterion for model evaluation. For example, in DeePEL, the DL-based model used to differentiate the transcription regulatory program between promoter-originated lncRNA (p-lncRNA) and enhancer-originated lncRNA (e-lncRNA), the authors mainly relied upon MCC as an evaluation metric since the dataset was imbalanced [57]. Additionally, instead of using cross-entropy loss, we may use weighted entropy loss, which penalizes the model for the misclassification of samples from the smaller class.

### 4.3. Interpreting and Visualizing Convolutional Networks

The interpretation of DL-based models is difficult [111]. Usually, DL-based models perform better than traditional ML-based models in terms of different evaluation metrics, which indicates that meaningful representations of data are learned by DL-based models. In terms of model explainability, the lowest-level (the level closest to the input data) representations are relatively simple to explain, but the higher-level features learned by different layers of DL-based models are difficult to interpret and can be considered to be a black box [112]. Opening this black box to interpret the high-level learned features will have a real impact on understanding the underlying biology of lncRNAs.

Feature importance scores can be used for the purpose of identifying the parts of an input that significantly contributed to achieving the result of the models. This can be done using two different methods: perturbation-based methods [113,114] and backpropagation-based methods [115,116]. For perturbation-based methods in sequence-based models, the input sequence is changed systematically (e.g., single-nucleotide substitution) to observe its impact on model performance. The main limitation of this approach is the high computational cost since we need to exhaustively search the perturbation. In backpropagation-based methods, the output signal is propagated backward from the output layer of the neural network to the input layer to check the contribution of different parts of the network. This approach is computationally more efficient and requires less time. For a more comprehensive discussion on model interpretability, readers may consult [117,118].

### 4.4. Model Selection and Model Building

There are many different types of DL architecture, and model selection is not a trivial task. The most commonly used network architectures are based on CNN and/or RNN. CNN architectures are mainly suited for high-dimensional data such as 2D images, 3D images, or higher numbers of genomic sequence data. RNN-based models can capture long-range dependencies from varying lengths of genomic sequence data. Sophisticated models can be developed by integrating multiple architectures into a novel architecture [109].

Determining the optimal structure of a deep network is also challenging. The optimal number of hidden layers and hidden units are problem-specific, and validation sets should be used to determine the optimal setup. More layers and hidden units in the neural network increase model complexity (number of representable functions), and discovering the local optimum becomes less prone to weight initialization [119].

**Figure 12:**
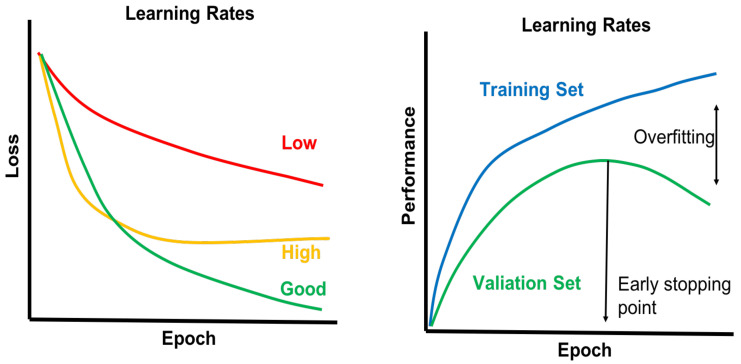
Loss function and performance metric over epoch to avoid the overfitting problem of deep networks. When the model performance of a validation set diminishes relative to the performance of a training set, an overfitting scenario may be indicated.

Training a deep network is far more complex and difficult than a shallow network [112]. Overfitting is a major challenge for training deep networks that result from using a model too complex for the data size of training sets. To avoid overfitting problems, the change of loss can be evaluated as a function of the number of epochs in the training phase. Depending on the learning rate value, the learning curve may change slowly or abruptly (Figure 12). Extreme learning rate values may result in a fluctuating learning curve [107]. Along with the loss function, monitoring the target performance parameter (e.g., accuracy, F1-score, etc.) is crucial for avoiding overfitting in both training sets and validation sets.

### 4.5. Confidence Score of the Prediction

In ML classification tasks, our main focus always revolves around the performance metric of the model. However, for real-life healthcare-related problems, we not only prefer a high prediction capability but also need to measure how confident the model is about its prediction, which enables us to evaluate the reliability of the model in clinical decision support systems, for example [120]. It is recommended that post-scaling be applied to Softmax output values from deep networks, as they are usually not on the right scale. Several methods have been proposed for the post-scaling purpose, such as temperate scaling [121], Platt scaling [122], isotonic regression [123], etc.

### 4.6. Catastrophic Forgetting

Catastrophic forgetting is a tendency of DL-based models to forget previously learned knowledge upon learning information from a new dataset [124]. Despite this, the integration of new lncRNA-related information is quite common, since new lncRNAs are constantly discovered and the information about known lncRNAs is increasing. For example, GENCODE release 21, published in 2014, contained 15,877 lncRNA genes. In 2019, this number increased to 17,904 lncRNA genes in GENCODE release 31. DL-based models that were developed based on earlier versions of data may not perform at the same level for newly released data. Training new models with new datasets are computationally exhaustive and time-consuming as well. There are different off-the-shelf solutions that may be used for this scenario such as dynamic neural networks with rehearsal training methods (e.g., Incremental Classifier and Representation Learning iCaRL [125]) and dual-memory-based learning systems [126].

## 5. Future Perspectives for Deep Learning in lncRNAome Research

DL-based methods are already extensively used in lncRNAs. However, to date, the most common DL architectures used in lnRNA-related research are CNN and RNN (see Table 1). Despite this, there are some other emerging architectures that may have applications in lncRNA-related research.

Di Lena et al. [127] applied deep spatio-temporal neural networks (DST-NNs) [128] using spatial features (e.g., protein secondary structures, orientation probabilities, and alignment probabilities) to determine protein structure predictions. Baldi et al. [129] applied multidimensional recurrent neural networks (MD-RNNs) [130] to amino acid sequences, the correlated profiles, and the secondary structures of proteins. Convolutional auto-encoders (CAEs) are designed to capitalize on the advantages of both CNN and AE to learn the hierarchical representation of data [131]. To the best of our knowledge, CAEs, MD-RNNs, and DST-NNs have not yet been used in the lncRNA domain.

Graph convolutional networks (GCN) have been successfully used in predicting different molecular attributes such as solubility, drug efficacy, etc. Recently, GCN and attention-based mechanisms have been used in lncRNA–disease prediction [65]. However, GCN, or attention-based mechanisms, have not been used in lncRNA–protein predictions thus far, and this might be an interesting area for further research.

GAN belongs to unsupervised learning methods, where the goal is to discover the underlying patterns from the data. GAN can also generate new sample data (e.g., sequences) with some variations. To date, the application of GAN is mainly focused on image processing [43]. However, as a relatively new method, the application of GAN is extremely limited in genomics. GAN models have been used to generate protein-coding DNA sequences [132] as well as for designing DNA probes for protein binding microarrays but have not been used in lncRNA research.

Capsule network models are a relatively new invention in the DL domain [133]. These models attempt to mimic the hierarchical representation of the human brain. Recently, capsule network models have been successfully used to classify brain tumor images [134]. However, capsule networks have not been used in any significant application in the lncRNA domain. LncRNAome might be an interesting area for capsule network-based research.

## 6. Conclusions

In this article, we summarized the contribution of DL in nine different lncRNAome research areas and highlighted the challenges DL-based researchers may face while developing models for lncRNAome. Comparative results from DL- and ML-based models highlight DL-based models’ superiority in different lncRNAome prediction tasks. Specifically, in the study of lncRNA identification, the distinction of transcription regulation programs for lncRNA, lncRNA–protein interaction prediction, and lncRNA–disease association prediction, DL-based models have outperformed the traditional ML-based models. Based on these results, there is significant potential for the application of DL-based techniques in lncRNAome. Unfortunately, only a few DL-based models for the task of lncRNA localization prediction, lncRNA–DNA interaction prediction, and the distinction of transcription regulation program for lncRNA exist. Researchers should consider focusing on developing new DL-based models in these areas which have received relatively little attention from the scientific community. However, the development of DL-based models for lncRNAome is a daunting task. Due to the low expression level and cell-/tissue-specific nature of lncRNA, DL-based model development may need to overcome the challenges of utilizing a relatively smaller dataset while building cell-/tissue-specific models. Additionally, the evolving annotations of lncRNAs from multiple research groups orchestrate another layer of complication in integrating newly discovered lncRNA into existing models. Thus, in spite of DL-based models achieving high-level prediction accuracy thus far, huge challenges in applying DL-based models in lncRNAome still exist. Leveraging state-of-the-art DL-based techniques while improving the existing ones, we expect to gain a better insight into lncRNAome in the near future.

## Figures and Tables

**Table 1:** List of deep learning (DL)-based architectures that have been employed to solve key questions in lncRNA research.

Research Area	Proposed DL Based Architecture	References
LncRNA Identification	CNN and RNN	LncRNAnet [52]
	DBN	LncADeep [53]
	Embedding vector, BLSTM, CNN	Liu et al. [54]
	DNN	DeepLNC [55]
Distinct transcription regulation of lncRNAs	CNN	DeepCNPP [56], DeePEL [57]
Functional annotation of lncRNAs	DNN	LncADeep [53]
Localization prediction	DNN	DeepLncRNA [58]
lncRNA–protein interaction	Stacked auto-encoder, Random forest	IPminer [59], RPI-SAN [60], BGFE [61]
	Stacked auto-encoder, CNN	RPITER [62]
LncRNA–miRNA interaction	GCN	GCLMI [63]
LncRNA–DNA interaction	GCN	[64]
LncRNA–disease association	GCN and AM	GCNLDA [65]
	CNN and AM	CNNLDA [66]
	DNN	NNLDA [67]
Cancer type classification	MLP, CNN, LSTM, DAE	[68]

AM: attention mechanism. BLSTM: bi-directional long short-term memory. CNN: convolutional neural network. DAE: deep autoencoder. DBN: deep belief network. DNN: deep neural network. GCN: graph convolutional network. LSTM: long short-term memory. MLP: multi-layer perceptron. RNN: recursive neural network.

**Table 2:** Overview of articles for lncRNA identification leveraging DL-based techniques.

	LncRNAnet [52]	LncADeep [53]	Liu et al. [54]	DeepLNC [55]
Publication Year	2018	2018	2019	2016
Species	Human and Mouse	Human and Mouse	Human and Mouse	Human
Data source used	GENCODE 25, Ensembl	GENCODE 24, Refseq	GENCODE 28, Refseq	LNCipedia 3.1, Refseq
Number of lncRNA considered for training	~21k (~21k) lncRNA transcripts from human (mouse)	~66k (~42k) full length lncRNA transcripts from human (mouse)	28k (~17k) lncRNA transcripts from human (mouse)	~80k lncRNA transcripts and ~100k mRNA transcripts
Performance metric	SN, SP, ACC, F1-Score, AUC	SN, SP, Hm	SN, SP, ACC, F1-Score, AUC	SN, SP, ACC, F1-Score, Precision
Metrics for comparison against traditional ML based model *	ACC:91.79 ^#^	Hm: 97.7 ^#^	ACC:96.4 ^#^	ACC: 98.07
Intriguing features from the proposed model	ORF length and ratio	ORF length and ratio, k-mer composition and hexamer score, position specific nucleotide frequency etc.	k-mer embedding	Solely based on k-mer patterns
Source code/Implementation	N/A	https://github.com/cyang235/LncADeep/	N/A	http://bioserver.iiita.ac.in/deeplnc

ACC: accuracy. AUC: area under the receiver operating characteristics curve. Hm: harmonic mean of sensitivity and specificity. MCC: Matthews correlation coefficient. N/A: not available. ORF: open reading frame. SN: sensitivity. SP: specificity. * Performance metrics that were highlighted in the original research article for comparing against traditional machine learning (ML)-based models. ^#^: Performance on humans.

**Table 3:** Overview of articles for demystifying transcription regulation of lncRNA leveraging DL-based techniques.

	DeepCNPP [56]	DeePEL [57]
Publication Year	2019	2019
Species	Human	Human
Data source used	Dataset from [75]	FANTOM CAT [17]
Number of lncRNA transcripts or genes considered	~19k lncRNA genes	~7k (~3k) p-lncRNA (e-lncRNA) transcripts
Performance metric	SN, SP, ACC	SN, SP, MCC, AUC
Metrics for comparison against traditional ML based model *	ACC: 83.34	Traditional ML model does not exist for this task
Intriguing features from the proposed model	k-mer embedding of promoter regions	k-mer embedding of promoter regions, transcription factor binding sites

* Performance metrics that were highlighted in the original research article for comparing against traditional ML-based models.

**Table 4:** Overview of articles for lncRNA–protein interaction prediction leveraging DL-based techniques.

	IPminer [59]	RPI-SAN [60]	BGFE [61]	RPITER [62]
Publication Year	2016	2018	2019	2019
Species	Multi-species	Multi-species	Multi-species	Multi-species
Benchmark Data source used	NPInter 2.0, RPI369, RPI488, RPI1807, RPI2241, RPI13254	NPInter 2.0, RPI488, RPI1807, RPI2241	RPI488, RPI1807, RPI2241	NPInter 2.0, RPI369, RPI488, RPI1807, RPI2241
Performance metric	SN, SP, ACC, Precision, AUC, MCC	SN, SP, ACC, Precision, AUC, MCC	SN, SP, ACC, Precision, AUC, MCC	SN, SP, ACC, Precision, AUC, MCC
Metrics for comparison against traditional ML based model for different dataset *	NPInter 2.0 (ACC: 95.7) ^#^, RPI369 (ACC: 75.2), RPI488 (ACC: 89.1), RPI1807 (ACC: 98.6), RPI2241 (ACC: 82.4), RPI13254 (ACC: 94.5)	NPInter 2.0 (ACC: 99.33) ^#^, RPI488 (ACC: 89.7), RPI1807 (ACC: 96.1), RPI2241 (ACC: 90.77)	RPI488 (ACC: 88.68), RPI1807 (ACC: 96.0), RPI2241 (ACC: 91.30)	NPInter 2.0 (ACC: 95.5) ^#^, RPI369 (ACC: 72.8), RPI488 (ACC: 89.3), RPI1807 (ACC: 96.8), RPI2241 (ACC: 89.0)
Intriguing features from the proposed model	Sequence composition features, specifically 3-mer and 4-mer from protein and RNA sequences, respectively	k-mer sparse matrix from RNA sequences and PSSM from protein sequences	k-mer sparse matrix from RNA sequences and PSSM from protein sequences. Stacked auto-encoder was employed to get high accuracy	k-mer frequency of sequence and two types of structural information (bracket and dot) from RNA. k-mer frequency of sequence and three types of structural information (α-helix, β-sheet and coil) from protein
Source code/Implementation	https://github.com/xypan1232/IPMiner; http://www.csbio.sjtu.edu.cn/bioinf/IPMiner	N/A	N/A	https://github.com/Pengeace/RPITER

PSSM: position-specific scoring matrix.* Performance metrics that were highlighted in the original research article for comparing against traditional machine learning (ML)-based models. ^#^: Performance on humans.

**Table 5:** Overview of articles for lncRNA–disease association prediction leveraging DL-based techniques.

	GCNLDA [65]	CNNLDA [66]	NNLDA [67]
Publication Year	2019	2019	2019
Data source used	LncRNADisease, Lnc2cancer, GeneRIF	LncRNADisease, Lnc2cancer, GeneRIF	LncRNADisease
Number of lncRNA considered	240	240	19166
Number of diseases considered	402	402	529
Performance metric	AUC, AUPRC, Precision, Recall	AUC, AUPRC, Precision, Recall	HR(k): Probability for the predicted samples to appear in top-k ranked list
Metrics for comparison against traditional ML based models	AUC ^$^: 0.959 AUPRC ^$^: 0.223	AUC ^$^: 0.952 AUPRC ^$^: 0.251	HR(k); k = 1.10
Intriguing features from the proposed model *	For ncRNA-lncRNA similarity Chen’s method was applied [105]. For disease-disease similarity Wang’s method was applied [106]	For ncRNA-lncRNA similarity Chen’s method was applied [105]. For disease-disease similarity Wang’s method was applied [106]	Matrix factorization method was modified in two aspects to fit into this model: (a) cross-entropy was used as a loss function; (b) only one batch data per round was used to minimize loss
Source code/Implementation	N/A	N/A	https://github.com/gao793583308/NNLDA

AUPRC: area under the precision-recall curve. HR(k): hit ratio, the probability for the predicted samples to appear in a top k ranked list. * Performance metrics that were highlighted in the original research article for comparing against traditional ML -based models. ^$^: Average over 402 diseases.
